# P2X7 Receptor Deficiency Ameliorates STZ-induced Cardiac Damage and Remodeling Through PKCβ and ERK

**DOI:** 10.3389/fcell.2021.692028

**Published:** 2021-07-29

**Authors:** Shanjun Huang, Weiqi Wang, Li Li, Ting Wang, Yihan Zhao, Ya Lin, Weijian Huang, Yonghua Wang, Zhouqing Huang

**Affiliations:** ^1^The Key Laboratory of Cardiovascular Disease of Wenzhou, Department of Cardiology, The First Affiliated Hospital of Wenzhou Medical University, Wenzhou, China; ^2^Department of Cardiology, The Fourth Affiliated Hospital, Zhejiang University School of Medicine, Yiwu, China; ^3^Department of Anesthesiology, The Fourth Affiliated Hospital, Zhejiang University School of Medicine, Yiwu, China; ^4^Department of Physical Education, Wenzhou Medical University, Wenzhou, China

**Keywords:** diabetic cardiomyopathy, P2X7 receptor, cardiac remodeling, PKCβ, ERK

## Abstract

Diabetic cardiomyopathy (DCM) is a complication of diabetes mellitus which result in cardiac remodeling and subsequent heart failure. However, the role of P2X7 receptor (P2X7R) in DCM has yet to be elucidated. The principal objective of this study was to investigate whether P2X7R participates in the pathogenesis of DCM. In this study, the C57BL/6 diabetic mouse model was treated with a P2X7R inhibitor (A438079). Cardiac dysfunction and remodeling were attenuated by the intraperitoneal injection of A438079 or P2X7R deficiency. *In vitro*, A438079 reduced high glucose (HG) induced cell damage in H9c2 cells and primary rat cardiomyocytes. Furthermore, HG/streptozotocin (STZ)-induced P2X7R activation mediated downstream protein kinase C-β (PKCβ) and extracellular regulated protein kinases (ERK) activation. This study provided evidence that P2X7R plays an important role in the pathogenesis of STZ-induced diabetic cardiac damage and remodeling through the PKCβ/ERK axis and suggested that P2X7R might be a potential target in the treatment of diabetic cardiomyopathy.

## Introduction

Many patients are diagnosed with diabetes worldwide, and the complications caused by diabetes are also diverse, such as diabetic nephropathy, diabetic retinopathy, and diabetic heart diseases. Among these complications, diabetic cardiomyopathy (DCM) is a serious complication that results in a poor prognosis for patients with diabetes (Zhao et al., 2012; Forbes and Cooper, 2013; Ofstad, 2016; Ogurtsova et al., 2017). In individuals with diabetes mellitus, a high blood glucose level is an independent causal factor for cardiomyopathy, one of the leading causes of hospitalization and death worldwide (Wang et al., 2006). DCM is characterized by structural and functional disorders, including ventricular dysfunction, interstitial fibrosis, cardiomyocyte hypertrophy, myocardial apoptosis, and metabolic deregulation (Wang et al., 2006). These pathophysiological changes eventually lead to cardiac remodeling and decreased cardiac output, preventing the heart from adequately pumping blood. Thus, an effective drug or target to treat DCM must be identified.

The P2X7 receptor (P2X7R), which is composed of 595 amino acids, is a non-selective cationic gated channel that assembles into a trimeric complex and belongs to the P2X family (Surprenant et al., 1996). After binding to extracellular ATP, the ion channel opens and allows K^+^, Na^+^, and Ca^2+^ plasma permeation (predominantly Ca^2+^) (Surprenant et al., 1996). The P2X7R is widely expressed *in vivo*, including nerve cells, liver cells, cardiomyocytes, monocytes, and macrophages (Burnstock and Knight, 2004). According to recent studies, P2X7R inhibition attenuates liver and lung fibrosis (Riteau et al., 2010; Huang et al., 2014). Simultaneously, P2X7R inhibition (using a siRNA or a pharmacological inhibitor) prevents the progression of atherosclerosis and myocardial infarction (Mezzaroma et al., 2011; Peng et al., 2015; Stachon et al., 2017). In diabetic animal models, the increase in P2X7R activity is involved in the pathogenesis of diabetic nephropathy (Kreft et al., 2016). P2X7Ri has been suggested to exert specific anti-inflammatory and anti-macrophage effects on diabetic nephropathy, ameliorating glomerular damage and fibrosis (Menzies et al., 2017). However, the role of the P2X7R in DCM has not been systematically determined.

The aim of the present study was to elucidate the essential role of the P2X7R in regulating DCM and its underlying mechanisms. The P2X7R-selective inhibitor A438079 was used in a streptozotocin (STZ)-induced type I diabetes mouse model, cultured H9c2 cells, and primary rat cardiomyocytes. The expression of P2X7R was significantly upregulated in the STZ-induced diabetic mouse model and high glucose (HG)-treated cell model. Moreover, P2X7R inhibition dramatically relieved cardiac damage by ameliorating fibrosis, apoptosis, and cardiomyocyte hypertrophy in STZ- or HG-induced models *in vivo* and *in vitro*. In addition, we further described the crucial role of P2X7R in knockout mice with diabetes.

## Materials and Methods

### Animal Experiments

Male C57BL/6 wild-type mice and male P2X7R**^–/–^** mice on a C57BL/6 background were provided by GemPharmatech Co., Ltd. (Nanjing, Jiangsu, China). Non-knockout male C57BL/6 mice were obtained from the Animal Center of Wenzhou Medical University. Animals were housed on a 12:12 h light–dark cycle at a constant room temperature and fed a standard rodent diet. The animals were acclimated to the laboratory for at least 2 weeks before initiating the studies. All operations were performed in accordance with the National Institutes of Health Guide for the Care and Use of Laboratory Animals. Animal care and experimental protocols were approved by the Committee on Animal Care of Wenzhou Medical University.

Eight- to twelve-week-old mice weighing 23–25 g were used to develop the diabetes mellitus model. Diabetes mellitus was induced by a single intraperitoneal (i.p.) injection of STZ (Sigma Chemicals, St. Louis, MO, United States) at a dose of 50 mg/kg formulated in 100 mM citrate buffer (pH 4.5) for 5 consecutive days. After 1 week, blood samples were collected. Fasting blood glucose levels were measured using a glucometer after a 12 h fast. Mice with fasting blood glucose levels >12 mmol/L were considered diabetic and chosen for further experiments. C57BL/6 wild-type mice and P2X7R**^–/–^** mice were randomly divided into four groups: (i) non-treated C57BL/6 control mice that received buffered saline (WT group, *n* = 7); (ii) non-treated P2X7R^–/–^control mice that received buffered saline (P2X7R**^–/–^** group, *n* = 7); (iii) STZ-induced diabetic mice (WT + STZ group, *n* = 7); and (iv) P2X7R**^–/–^** mice that received an i.p. injection of STZ (STZ + P2X7R**^–/–^** group, *n* = 7). Non-knockout male C57BL/6 mice were randomly divided into four groups: (I) non-treated C57BL/6 control mice that received buffered saline (Ctrl group, *n* = 8); (II) STZ-induced diabetic mice without treatment (STZ group, *n* = 8); (III) STZ-induced diabetic mice that received an i.p. injection of 10 mg/kg A438079 every second day for 16 weeks (STZ + A-10MG group, *n* = 8); and (IV) STZ-induced diabetic mice that received an i.p. injection of 20 mg/kg A438079 every second day for 16 weeks (STZ + A-20MG group, *n* = 9). All animals were sacrificed by administering sodium pentobarbital anesthesia. The hearts were collected and weighed. In addition, heart tissues were placed in 4% paraformaldehyde for the pathological analysis and/or snap-frozen in liquid nitrogen for gene and protein expression analyses.

### Cardiac Function

Systolic and diastolic cardiac function were determined non-invasively using transthoracic echocardiography in mice 2 h before sacrifice. Diastolic function was assessed by performing pulsed-wave Doppler imaging of the transmitral filling pattern. The ejection fraction (EF) was calculated from the left ventricle end-diastolic volume (LVEDV) and end-systolic volume (LVESV) using the following equation: (LVEDV−LVESV)/LVEDV× 100%. Fractional shortening (FS) was calculated using the following equation: FS = [(LVIDd−LVIDs)/LVIDd]×100%. The Tei index was determined based on the Doppler recordings of the left ventricular isovolumetric relaxation time (IRT), isovolumetric contraction time (ICT), and ejection time (ET):Tei=(IRT+ICT)/ET.

### Isolation of Primary Rat Cardiomyocytes

Newborn (2–3 days old) SD rats were obtained from the Animal Center of Wenzhou Medical University. The incision on the sternal left margin was disinfected with 75% alcohol. The hearts were removed from the chest, washed twice with cold PBS, and gently dissected. The heart fragments were digested with 0.08% trypsin for 8 min at 37°C with magnetic stirring. This step was repeated approximately 16 times until the tissue organization was no longer visible. The supernatant was collected, and the digestion was terminated by an incubation with DMEM containing 10% FBS. All the collected liquid was centrifuged at 1000 rpm for 10 min. The cells were incubated for 1 h at 37°C after suspension. The non-adherent cells were primary cardiomyocytes, and the adhesive cells were fibroblasts, which were all collected and cultivated for the experiment.

### H9c2 Cell and Primary Cardiomyocyte Culture and Treatment

The rat myocardium-derived cell line H9c2 was obtained from the Shanghai Institute of Biochemistry and Cell Biology (Shanghai, China). H9c2 cells or primary cardiomyocytes were maintained in DMEM (Gibco, Eggenstein, Germany) containing 5.5 mmol/L D-glucose, 10% FBS, 100 U/ml penicillin, and 100 mg/ml streptomycin at 37°C in a 5% CO_2_ incubator (Thermo Fisher Scientific, Waltham, MA, United States). Experiments were performed when the cell density is about 70–80%. In the HG-treated group, H9c2 cells or primary cardiomyocytes were incubated with DMEM containing 33 mmol/L glucose. A438079, LY317615, and PD98095 were dissolved in DMSO and added to the cells for 1 h. The final concentration of the three inhibitors was 10 μM. Afterward, the cells were exposed to HG for 24 h.

### Reagents

The P2X7R inhibitor (A438079), PKC inhibitor (LY317615), and ERK-specific inhibitor (PD98095) were purchased from Selleck (Shanghai, China). A RevertAid First Strand cDNA Synthesis Kit was obtained from Thermo Fisher Scientific (Waltham, MA, United States). SYBR Premix Ex Taq^TM^ (TliRnaseH Plus) was obtained from Takara (Dalian, China). The primary anti-P2X7R antibody (#APR-004) was obtained from Alomone Labs (Israel). Anti-collagen 1 (anti-COL-1, ab34710), anti-matrix metalloproteinase-9 (anti-MMP9, ab38898), and anti-heavy chain cardiac myosin (ab185967) antibodies were obtained from Abcam (Cambridge, United Kingdom). Moreover, antibodies against TGFβ (#3711), GAPDH (#5174), Caspase-3 (#9662), Bcl2 (#2870), phospho-PKCβII (Thr638/641) (#9375), Bax (#5023), and Phospho-p44/42 MAPK (ERK1/2) (Thr202/Tyr204; #4370) were obtained from Cell Signaling Technology (Danvers, MA, United States).

### Western Blot Analysis

Protein isolation and western blot analyses were performed using methods described in the literature (Huang et al., 2008). Protein samples were loaded and separated on SDS-PAGE gels before being transferred to a PVDF membrane (Millipore, MA, United States). Membranes were blocked with a 5% fat-free milk solution in TBST (TBS containing 0.1% Tween-20) at room temperature for 1 h, followed by an overnight incubation at 4°C with the appropriate primary antibodies. Immunoreactive bands were incubated with a secondary antibody at room temperature for 1 h and labeled with horseradish peroxidase after three washes. Proteins were detected with the ECL reagent (Bio-Rad, United States).

### Real-Time Quantitative PCR

Total RNA was extracted from H9c2 cells or heart tissues (20–50 mg) using TRIzol reagent according to the manufacturer’s protocol (Invitrogen Life Technologies). One microgram of total RNA from each sample was used to generate cDNAs using the RevertAid First Strand cDNA Synthesis Kit (#K1622; Thermo Fisher Scientific). The resulting cDNA templates were amplified using SYBR in a real-time polymerase chain reaction with primers from Sangon Biotech (Shanghai, China) (Table 1). PCR was directly monitored using CFX 96 (Bio-Rad, United States). All results were normalized to GAPDH.

**TABLE 1:** The primer sequences of each target gene.

Gene	Species	FW	RW
P2X7R	Rat	CGGGCCACAACTATACTACGA	CCTGAACTGCCACCTCTGTAA
TGF-β	Rat	GACTCTCCACCTGCAAGACC	GGACTGGCGAGCCTTAGTTT
ANP	Rat	GTACAGTGCGGTGTCCAACA	ATCCTGTCAATCCTACCCCC
MyHC	Rat	GAACACCAGCCTCATCAACC	CCTTCTTGGCCTTCTCCTCT
GAPDH	Rat	ACAGCAACAGGGTGGTGGAC	TTTGAGGGTGCAGAGAACTT
P2X7R	Mouse	AGAATGAGTTCCCCTGCAAA	AAGCTGTACCAGCGGAAAGA
TGF-β	Mouse	TGACGTCACTGGAGTTGTACGG	GGTTCATGTCxATGGATGGTGC
ANP	Mouse	AAGAACCTGCTAGACCACCTGGAG	TGCTTCCTCAGTCTGCTCACTCAG
MyHC	Mouse	CAAAGGCAAGGCAAAGAAAG	TCACCCCTGGAGACTTTGTC
GAPDH	Mouse	ACCCAGAAGACTGTGGATGG	TTCAGCTCAGGGATGACCTT

### Terminal Deoxynucleotidyl Transferase-Mediated dUTP Nick End Labeling Staining

Paraffin tissue sections with a thickness of 5 μm were used for terminal deoxynucleotidyl transferase-mediated dUTP nick end labeling (TUNEL) staining of apoptotic cells using a One-Step TUNEL Apoptosis Assay Kit from Beyotime (Shanghai, China) according to the manufacturer’s instructions. TUNEL-positive cells were imaged under a fluorescence microscope (400× amplification; Nikon, Japan).

## Histopathological Masson’s Trichrome, Sirius Red and Rhodamine Staining

Excised heart tissue specimens were fixed with 4% paraformaldehyde, processed in a graded series of alcohol and xylene solutions and then embedded in paraffin. Paraffin blocks were sliced into sections at a thickness of 5 μm. After rehydration, the sections were stained with hematoxylin and eosin (H&E). Images of each section were captured using a light microscope (400× amplification; Nikon, Japan) to evaluate the histopathological damage. Paraffin sections (5 μm) were stained with 0.1% Sirius Red and Masson’s trichrome (Sigma) to evaluate the level of collagen deposition and fibrosis, respectively. H9c2 cells were pretreated with HG (33 mM) for 24 h in the presence or absence of A438079. Then, the cells were stained with rhodamine (Beyotime; Shanghai, China) according to the manufacturer’s instructions. The stained sections or cells were then viewed under a Nikon fluorescence microscope (400× amplification; Nikon, Japan).

### Flow Cytometry

H9c2 cells were pretreated with HG (33 mM) for 24 h before the level of apoptosis was measures using flow cytometry. An Annexin V-EGFP/PI Cell Apoptosis Detection Kit (KeyGen Biotech, Nanjing, China) was used for this experiment. Then, 0.25% trypsin without EDTA was used to digest cells, and the cells were centrifuged at 2000 rpm for 5 min. Pellets were washed twice with PBS. Binding buffer (500 μl) was added to suspend the cell pellets. After mixing the cells with 5 μl of Annexin V-FITC and 5 μl of propidium iodide and reacting for 5–15 min lucifugally, the cells were immediately analyzed using a FACSCalibur flow cytometer (Becton Dickinson & Co., United States).

### Statistical Analysis

All data were obtained from three independent experiments and are presented as the means ± SEM. All statistical analyses were performed using GraphPad Pro Prism 5.01 software (GraphPad, San Diego, CA, United States). One-way ANOVA followed by the multiple comparisons test with the Bonferroni correction were employed to analyze the differences between sets of data. A *p*-value < 0.05 was considered statistically significant.

## Results

### P2X7 Receptor Expression Was Substantially Increased in the STZ-Induced Type 1 Diabetes Model and HG-Treated Cell Model *in vitro*

**FIGURE 1:**
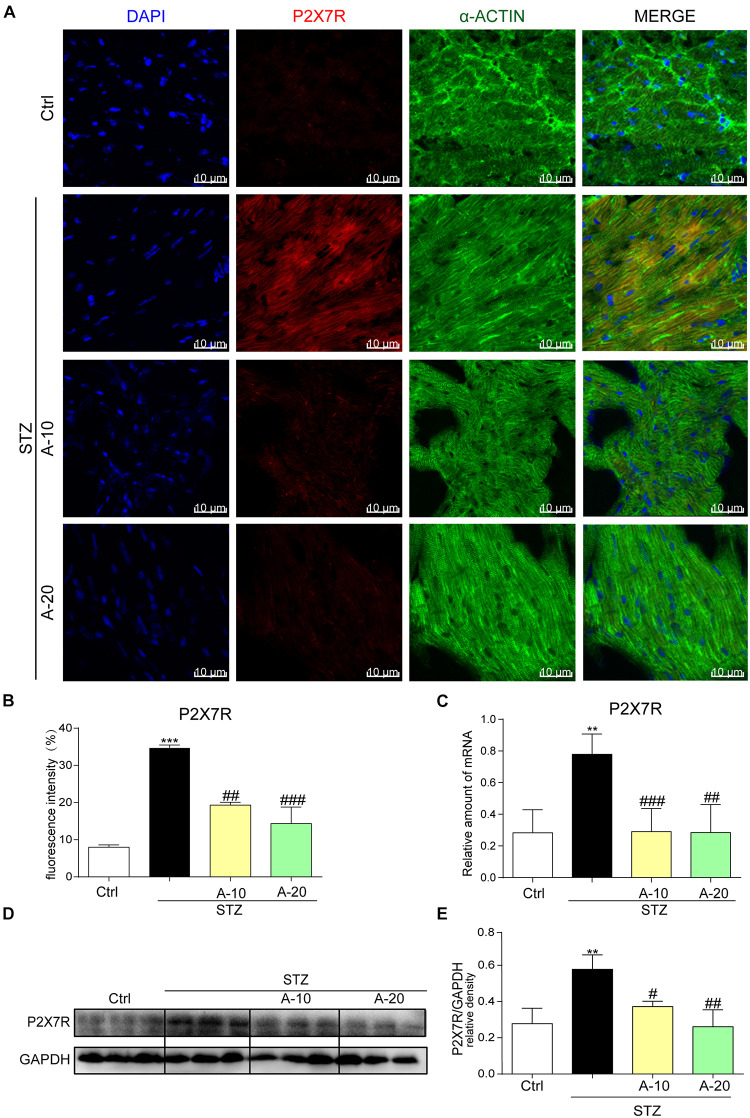
P2X7 receptor expression was increased in the STZ-induced type 1 diabetes model and HG-treated cell model *in vitro*. Representative images **(A)** and quantification **(B)** of immunofluorescence staining for P2X7R in myocardial tissues (400× magnification). The expression of the P2X7R mRNA **(C)** and protein **(D)** with the corresponding statistics **(E)** (data from three independent experiments were analyzed: *p < 0.05, **p < 0.01, and ***p < 0.001 compared with the Ctrl; ^#^*p* < 0.05, ^##^*p* < 0.01, and ^###^*p* < 0.001 compared with STZ alone).

In the diabetic mouse model induced by STZ, immunofluorescence staining was utilized to inspect P2X7R expression in the myocardial tissue of C57BL/6 mice (Figure 1A). The expression of P2X7R was significantly increased after the administration of STZ. However, the administration of A438079, a selective inhibitor of the P2X7R, significantly reduced the fluorescence intensity. Moreover, compared with the low-dose group (10 mg/kg), the decreasing trend observed in the high-dose group (20 mg/kg) was more evident based on the fluorescence intensity (Figure 1B). As expected, the results obtained for the protein and mRNA levels were consistent with the immunofluorescence double staining (Figures 1C–E). Collectively, the expression of the P2X7R was upregulated in the STZ-induced diabetic model.

We used HG (33 mM) to stimulate H9c2 cells at different time points and evaluate whether the upregulation of the P2X7R *in vitro* was consistent with the changes observed *in vivo*. As shown in Supplementary Figures 1A,B, P2X7R levels increased and leveled off after 24 h. Thus, we chose 24 h as the time point for our subsequent experiments. In H9c2 cells and primary rat cardiomyocytes pretreated with A438079 for 1 h, P2X7R expression was significantly reduced at both the protein and mRNA levels (Supplementary Figures 1C,D). Interestingly, in primary fibroblasts, the expression of the P2X7R was not altered after HG stimulation (Supplementary Figures 1E,F). Based on this phenomenon, HG mainly altered the level of the P2X7R at the myocardial cell surface.

### P2X7 Receptor Inhibition Alleviated STZ-Induced Cardiac Dysfunction

We performed additional experiments to detect the effect of P2X7R inhibition on the status and cardiac function of mice. The weight of C57BL/6 mice was decreased and fasting blood glucose levels increased significantly after the administration of STZ (Supplementary Figures 1G,H). However, a slight increase in body weight was detected in A438079-treated mice, and no change in fasting plasma glucose levels was observed (Supplementary Figures 1G,H) after treatment with A438079.

Non-invasive transthoracic echocardiography was used to examine the cardiac function of all experimental mice 2 h before sacrifice (Table 2). Echocardiography data revealed that the heart rate was not affected. Heart weight/body weight (HW/BW) was significantly increased in the STZ group compared with the other groups. Furthermore, STZ not only disrupted diastolic function (as observed in the IVSd, LVIDd, IRT, and Tei indices) but also reduced contraction function (EF% and FS%). As expected, these dysfunctions were substantially attenuated by the A438079 treatment.

**TABLE 2:** Biometric and echocardiographic parameters of the C57BL/6 experimental mice.

	Ctrl (*n* = 8)	STZ		
		*n* = 8	A-10MG (*n* = 8)	A-20MG (*n* = 9)
HR, bpm	525 ± 79	489 ± 78	514 ± 54	527 ± 41
HW/BW, mg/g	5.78 ± 0.26	6.61 ± 0.36***	5.74 ± 0.34^###^	5.94 ± 0.23^##^
IVSD, mm	0.73 ± 0.04	0.75 ± 0.08	0.73 ± 0.03	0.70 ± 0.03
LVIDd, mm	2.23 ± 0.10	2.45 ± 0.06	2.17 ± 0.15^#^	2.16 ± 0.12^##^
IRT, ms	14.14 ± 2.03	24.5 ± 1.91***	16.67 ± 1.37	17.33 ± 2.18^###^
Tei index	0.70 ± 0.07	0.84 ± 0.14	0.73 ± 0.02	0.70 ± 0.02
EF%	82.03 ± 2.94	73.26 ± 1.45**	79.58 ± 2.11^#^	79.26 ± 1.86^#^
FS%	44.09 ± 2.37	36.85 ± 2.25**	43.36 ± 3.33^#^	42.99 ± 3.22^#^

*Ctrl, control; HR, heart rate; HW/BW, heart weight/body weight; IVSD, diastole interventricular septal thickness; LVIDd, diastole left ventricle internal dimension; IRT, isovolumic relaxation time; EF%, ejection fraction %; FS%, fractional shortening %.*

*Data presented as Mean ± SD, ^∗∗^*p* < 0.01, and^∗∗∗^*p* < 0.001, compared to Ctrl group; ^#^*p* < 0.05, ^##^*p* < 0.01, and ^###^*p* < 0.001, compared to STZ group.*

### Blockade of the P2X7 Receptor Attenuated STZ-Induced Cardiac Remodeling and Apoptosis *in vivo*

**FIGURE 2:**
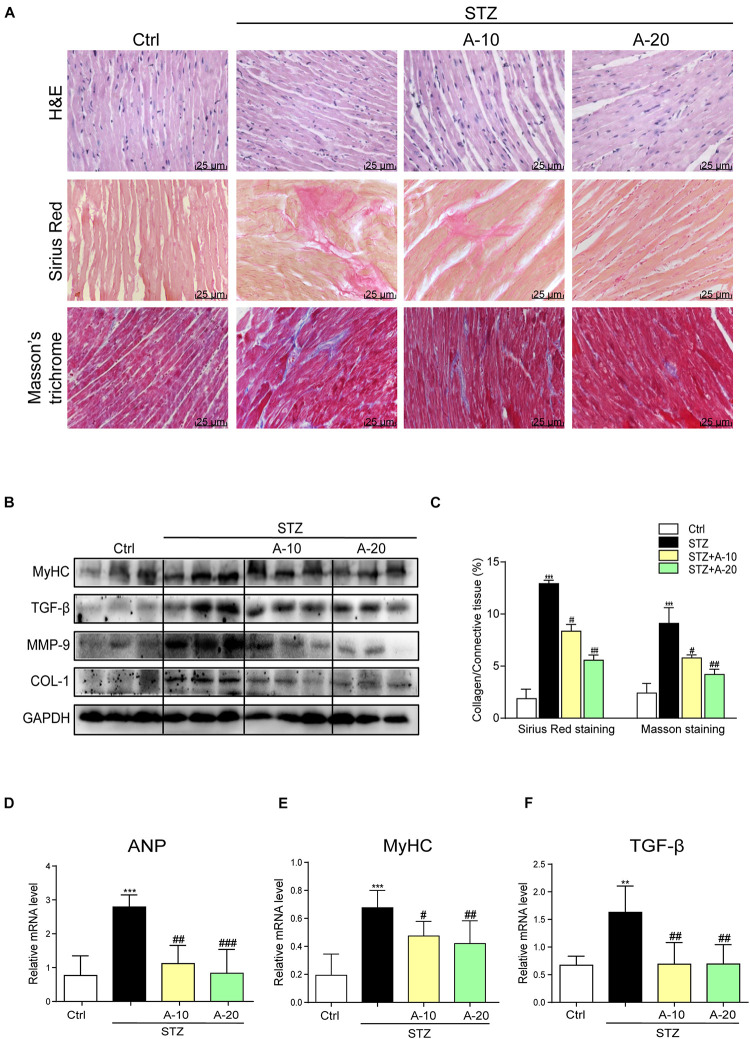
Blockade of the P2X7 receptor attenuated STZ-induced cardiac remodeling. Representative images of hematoxylin-eosin staining (H&E) of the myocardial tissues (400× magnification), (**A**, upper panel). Myocardial fibrosis analysis was detected using Sirius Red staining (**A**, middle panel), and representative images of Masson’s trichrome staining (**A**, bottom panel) are shown (400× magnification). Bar graph showing the quantified interstitial fibrotic areas (%) in images of Sirius Red staining and Masson’s trichrome staining **(C)**. Levels of the MyHC, TGF-β, MMP-9, and COL-1 proteins in myocardial tissues were measured using western blotting **(B)** (*n* = 3 per group). The mRNA expression of the hypertrophy markers MyHC and ANP **(D,E)** and fibrosis marker TGF-β **(F)** in the myocardial tissues is shown (data from three independent experiments were analyzed: *p < 0.05, **p < 0.01, and ***p < 0.001 compared with the Ctrl; ^#^*p* < 0.05, ^##^*p* < 0.01, and ^###^*p* < 0.001 compared with STZ alone).

Although A438079 improved the cardiac function of diabetic mice, we were not sure whether it improved cardiac remodeling. Thus, H&E staining was used to detect the structural morphology. The hearts of STZ-challenged mice displayed structural abnormalities, such as disorganized myofibers. This disorder was improved in the other groups treated with the inhibitor (Figure 2A).

Fibrosis is also an important pathological variation observed in individuals with DCM (Wang et al., 2006). The connective tissue in the myocardium was examined using Masson’s trichrome and Sirius Red staining for collagen (Figure 2A). The hearts from the STZ group showed apparent collagen and fibrous tissue accumulation, which were suppressed by treatment with A438079 (Figures 2A,C). At the protein level, the expression of the profibrotic markers TGF-β, COL-1, and MMP-9 was increased in the hearts of STZ-induced diabetic mice (Figure 2B). The results of PCR for TGF-β showed the same trend (Figure 2F). These molecular biological changes were remarkably reversed by A438079 administration (Figures 2B,F). Moreover, higher expression of myosin heavy chain (MyHC), a biomarker of cardiac hypertrophy, was observed in the STZ-induced group than in the A438079 treatment groups (Figures 2B,E). Similar results were observed for the expression of the ANP mRNA (Figure 2D). Taken together, P2X7R inhibition significantly improved myocardial remodeling.

**FIGURE 3:**
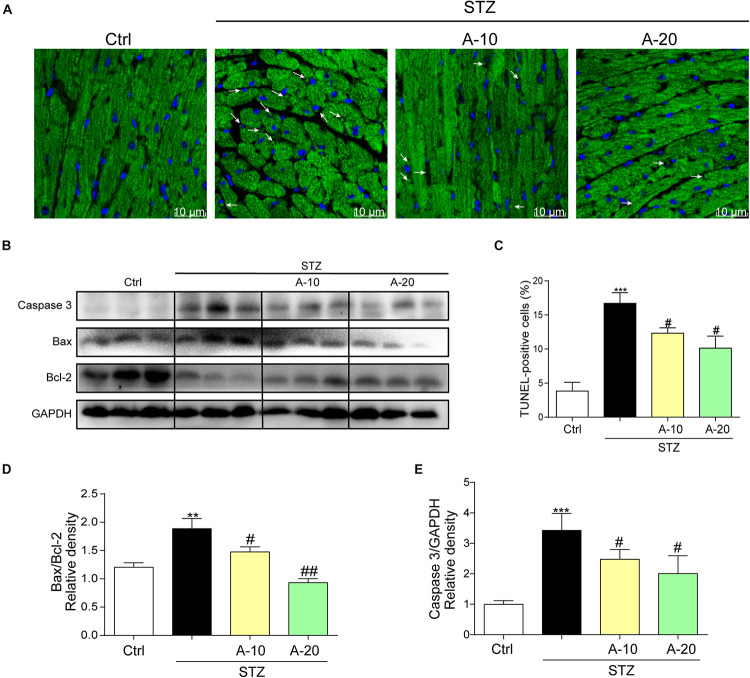
Blockade of the P2X7 receptor improved STZ-induced cardiac apoptosis. Representative images **(A)** and quantification **(C)** of TUNEL staining in mouse myocardial tissues are shown (400× magnification). Levels of the apoptosis-related proteins Caspase-3, Bcl-2, and Bax were measured by western blot **(B)**. (*n* = 3 per group). The ratio of the Bcl-2 protein to the Bax protein and the ratio of Caspase3/GAPDH proteins are shown **(D,E)** (data from three independent experiments were analyzed: *p < 0.05, **p < 0.01, and ***p < 0.001 compared with the Ctrl; ^#^*p* < 0.05, ^##^*p* < 0.01, and ^###^*p* < 0.001 compared with STZ alone).

In addition, apoptosis plays an important role in DCM. As shown in images of TUNEL staining, A438079 played a role in preserving myocardial cell survival and reducing apoptosis caused by diabetes (Figures 3A,C). The levels of apoptosis-related proteins, such as Caspase-3 and Bax, were increased due to diabetes, and the level of the antiapoptotic protein Bcl-2 was decreased (Figures 3B,E). P2X7R inhibition led to changes in the levels of apoptotic proteins, and the Bax-to-Bcl-2 ratio was decreased (Figure 3D).

### P2X7 Receptor Inhibition Prevented HG-Induced Phenotypic Changes in H9c2 Cells and Primary Rat Cardiomyocytes

**FIGURE 4:**
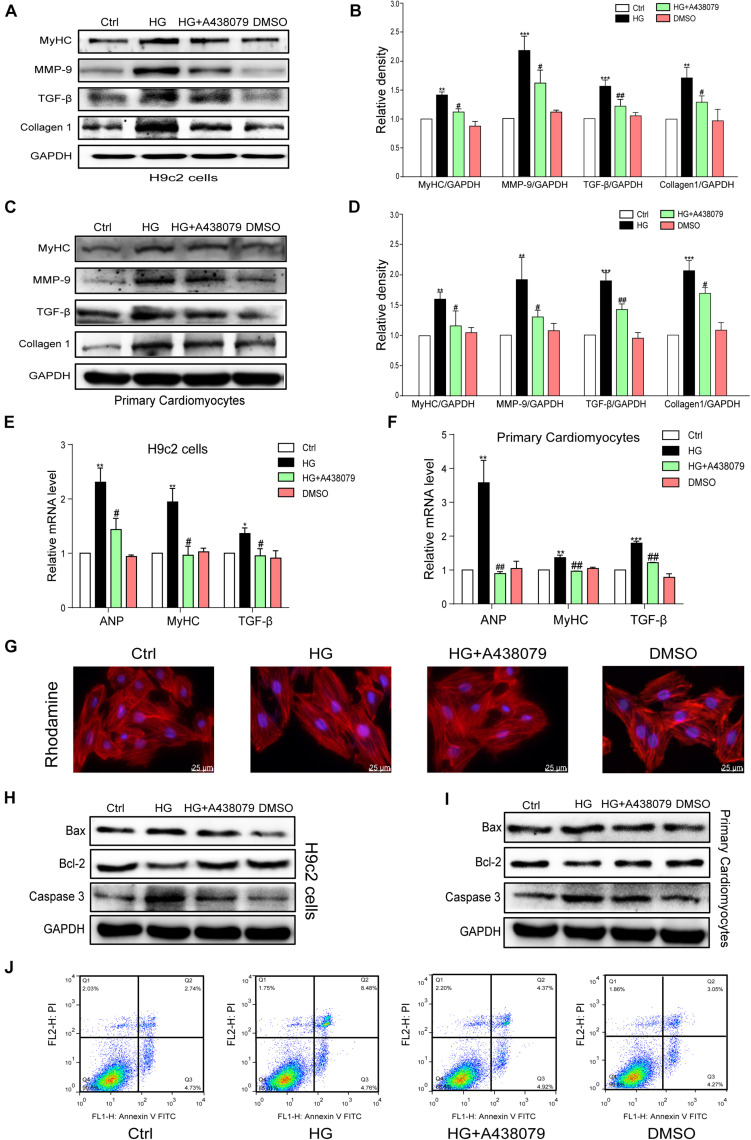
P2X7 receptor inhibition prevented HG-induced phenotypic changes and apoptosis in H9c2 cells and rat primary cardiomyocytes. The levels of pro-fibrotic (MMP-9, TGF-β, and COL-1) and pro-hypertrophic proteins (MyHC) was examined using western blot analysis **(A,B)**. **(E)** The bar graph shows the corresponding PCR data for TGF-β, MYHC, and ANP. The same process and analysis were performed for rat primary cardiac myocytes. (WB: **C,D**, PCR: **F**). Representative images of rhodamine staining in each group of H9c2 cells **(G)**. The levels of the apoptosis-related proteins Caspase-3, Bcl-2, and Bax were measured on H9c2 cells and primary cardiac myocytes using western blotting **(H,I)**. Flow cytometry analysis showing that A438079 reduced the apoptosis of HG-treated H9c2 cells **(J)**. (Data from three independent experiments were analyzed: *p < 0.05, **p < 0.01, and ***p < 0.001 compared with the Ctrl; ^#^*p* < 0.05, ^##^*p* < 0.01, and ^###^*p* < 0.001 compared with STZ alone).

The tests described below were performed to confirm whether P2X7R inhibition using A438079 protected the myocardium *in vitro*. H9c2 cells and primary rat cardiomyocytes were incubated with A438079 for 1 h prior to HG (33 mM) stimulation for 24 h. The protein levels of the hypertrophic marker MyHC and profibrotic proteins TGF-β, MMP-9, and COL-1 in the respective cells were remarkably decreased by the A438079 pretreatment (Figures 4A–D). The results of quantitative PCR showed that P2X7R inhibition alleviated HG-induced hypertrophy and fibrosis to varying degrees (Figures 4E,F). As intuitively observed from the rhodamine staining, the inhibition of the P2X7R improved the hypertrophic response of H9c2 cells to HG (Figure 4G). These phenotypic changes were consistent with the results obtained from the hearts of STZ-induced diabetic mice.

### Pharmacological Inhibition of the P2X7 Receptor Attenuated HG-Induced Apoptosis in H9c2 Cells and Rat Primary Cardiomyocytes

A selective inhibitor of the P2X7R was used *in vitro* to confirm the role of the P2X7R in HG-induced apoptosis. H9c2 cells and rat primary cardiomyocytes were pretreated with A438079 for 1 h and then exposed to HG (33 mM) for 24 h. As shown in Figures 4H,I, the expression of Bax and Caspase-3 was significantly increased in the HG group but was obviously downregulated by the A438079 treatment in H9c2 cells and rat primary cardiomyocytes. Similar trends in the ratio of Bax to Bcl-2 and the ratio of Caspase-3 to GAPDH were also observed (Supplementary Figures 2A–D). The flow cytometry results for H9c2 cells revealed a higher percentage of apoptotic cells in the HG stimulation group than in the normal group. In addition, the A438079 treatment significantly suppressed apoptosis, particularly late apoptosis (Figure 4J).

### P2X7 Receptor Knockout Improved STZ-Induced Cardiac Dysfunction

**FIGURE 5:**
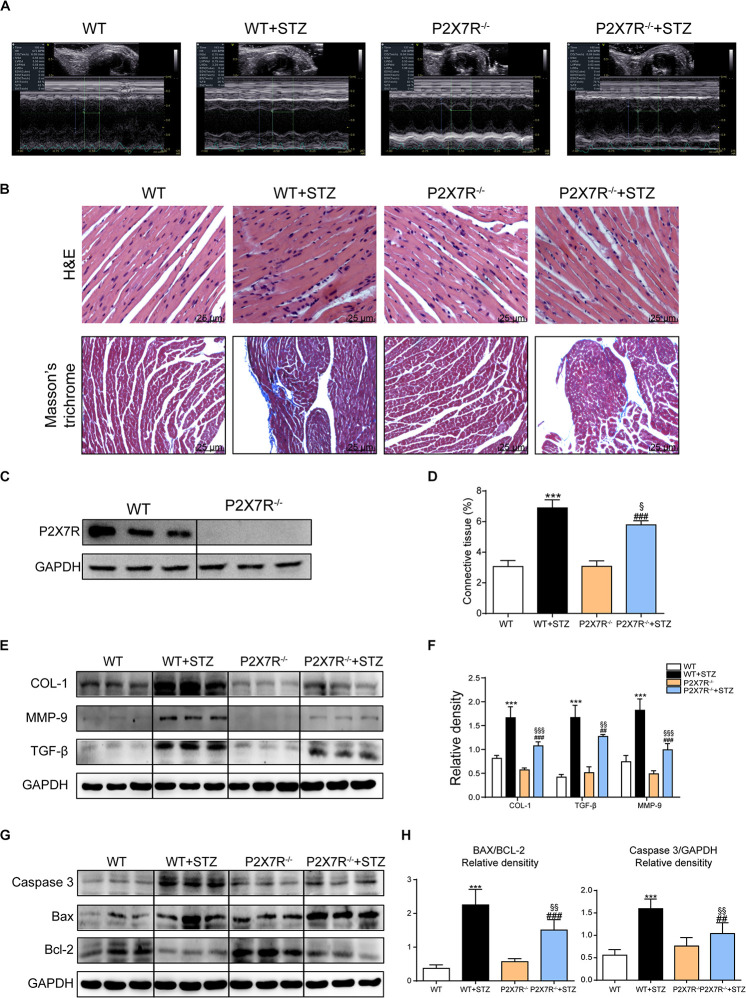
The P2X7 receptor was involved in STZ-induced cardiac damage. Representative images **(A)** of echocardiograms from each group of knockout mice. Representative images of hematoxylin-eosin staining (H&E) of the myocardial tissues from knockout mice (400× magnification), (**B**, upper panel). Myocardial fibrosis was analyzed using Masson’s trichrome staining, and representative images (**B**, bottom panel) are shown (400× magnification). Quantification of the interstitial fibrotic areas (%) in images of Masson’s trichrome staining are shown in the bar graph **(D)**. The expression of the P2X7 receptor in myocardial tissues from knockout mice **(C)**. The protein levels of fibrosis markers in myocardial tissues were measured using western blotting **(E,F)** (*n* = 3 animals per group). The levels of the apoptosis-related proteins Caspase-3, Bcl-2, and Bax were measured using western blotting **(G)**. The ratio of the Bcl-2 protein to the Bax protein and the ratio of Caspase3/GAPDH proteins are shown **(H)**. (Data from three independent experiments were analyzed: *p < 0.05, **p < 0.01, and ***p < 0.001 compared with WT mice; ^#^*p* < 0.05, ^##^*p* < 0.01, and ^###^*p* < 0.001 compared with P2X7R–/– mice; ^§^*p* < 0.05, ^§§^*p* < 0.01, and ^§§§^*p* < 0.001 compared to the WT + STZ group).

Based on previous animal experiments and *in vitro* experiments, pharmacological inhibition of the myocardial P2X7R reduced cardiac remodeling and apoptosis in the STZ-induced model or HG-treated cells. P2X7R knockout mice (P2X7R**^–/–^**) were used as experimental models to better clarify its role. Figure 5C shows the expression of the P2X7R in cardiac tissue from P2X7R**^–/–^** mice.

All mice were subjected to echocardiography 2 h before sacrifice. A significant difference in the heart rate was not observed among the four groups. Under basal conditions, P2X7R knockout alone had no effect on cardiac function (Figure 5A and Table 3). As shown in Table 3, STZ caused systolic dysfunction (as evidenced by the EF% and FS% indices). As expected, these dysfunctions improved in P2X7R**^–/–^** mice. Figure 5A shows a representative echocardiogram of each group, which presents the findings more intuitively.

**TABLE 3:** Biometric and echocardiographic parameters of the C57BL/6 gene knockout experimental mice.

	WT	WT + STZ	P2X7R^–/–^	P2X7R^–/–^ + STZ
HR, bpm	460 ± 41	400 ± 63	460 ± 67	418 ± 54
HW/BW, mg/g	5.21 ± 0.27	6.59 ± 0.56**	5.38 ± 0.33	6.07 ± 0.58^#^
IVSD, mm	0.63 ± 0.04	0.64 ± 0.03	0.63 ± 0.08	0.63 ± 0.05
LVIDd, mm	3.55 ± 0.32	3.83 ± 0.24*	3.40 ± 0.36	3.60 ± 0.18^#^^§§^
LVPWd, mm	0.62 ± 0.06	0.69 ± 0.05*	0.63 ± 0.04	0.67 ± 0.06^#^
LVIDs, mm	2.23 ± 0.27	2.50 ± 0.35*	2.15 ± 0.25	2.39 ± 0.20^##^
EF%	76 ± 5.94	62 ± 7.59***	77.96 ± 4.85	71.23 ± 3.91^###^^§§§^
FS%	39.23 ± 5.26	28.8 ± 5.35***	40.63 ± 4.68	35.52 ± 2.66^###^^§§§^

*HR, heart rate; HW/BW, heart weight/body weight; IVSD, diastole interventricular septal thickness; LVIDd, diastole left ventricle internal dimension; LVPWd, left ventricular diastole posterior wall thickness; LVIDs, systole left ventricle internal dimension; EF%, ejection fraction %; FS%, fractional shortening %.*

*Data presented as Mean ± SD, ^∗^*p* < 0.05, ^∗∗^*p* < 0.01, and ^∗∗∗^*p* < 0.001, compared to WT; ^#^*p* < 0.05, ^##^*p* < 0.01, and ^###^*p* < 0.001, compared to P2X7R^–/–^; ^§§^*p* < 0.01, and ^§§§^*p* < 0.001, compared to WT + STZ.*

### The P2X7 Receptor Was Involved in STZ-Induced Cardiac Remodeling and Apoptosis

P2X7R knockout mice were transformed into diabetic mouse models by administering them STZ, and then myocardial tissues were observed using histochemical staining. HE staining revealed that STZ-induced cardiomyocyte hypertrophy and histopathological alterations were alleviated by P2X7R knockout (Figure 5B). Masson’s trichrome staining showed that P2X7R**^–/–^** decreased cardiac fibrosis in STZ-induced mice (Figures 5B,D).

Strikingly, STZ-induced changes in the expression of the profibrotic proteins collagen I, MMP 9, and TGF-β were reversed in the P2X7R**^–/–^** mouse hearts (Figures 5E,F). Myocardial tissue apoptosis is shown in Figures 5G,H. The STZ treatment induced Caspase 3 and Bax expression and inhibited Bcl-2 expression. As expected, these abnormal changes were ameliorated by knockout of the P2X7R (Figures 5G,H). Based on these results, P2X7R enhanced STZ-induced remodeling and apoptosis in the mouse myocardial tissue.

### P2X7R Regulated the Activation of the PKCβ/ERK Axis in High Glucose-Induced Cardiomyocytes

**FIGURE 6:**
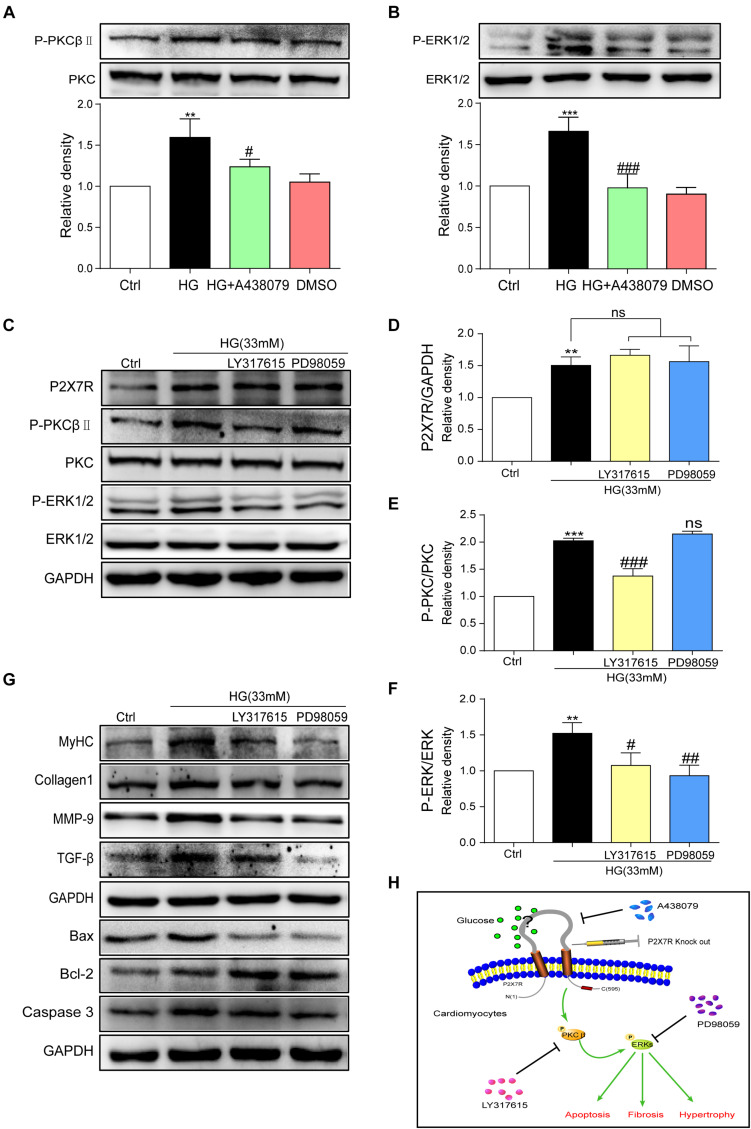
P2X7R regulated the activation of the PKCβ/ERK pathway in high glucose-induced cardiomyocytes. Total protein was extracted and p-PKC/PKC and p-ERK/ERK levels were analyzed using western blot analyses **(A,B)**. Levels of the P2X7R, p-PKC/PKC and p-ERK/ERK proteins were analyzed using western blotting **(C)**, and the quantitative statistics are presented in graphs **(D–F)**. The levels of pro-fibrotic, pro-apoptotic, and pro-hypertrophic proteins were determined using western blot analyses **(G)**. A schematic illustrating the role of P2X7R in diabetes/HG-induced injury in cardiomyocytes, the preventative effect of A438079 and the role of P2X7R knockout. **(H)** (Data from three independent experiments were analyzed: *p < 0.05, **p < 0.01, and ***p < 0.001 compared with the Ctrl; ^#^*p* < 0.05, ^##^*p* < 0.01, and ^###^*p* < 0.001 compared with HG alone).

The underlying mechanism by which HG causes damage must be elucidated. By reviewing previous studies, the PKC/MAPK pathway participates in the development of DCM (Soetikno et al., 2012). A PKC phosphorylation site was identified in the short N terminus of P2X7R (Boue-Grabot et al., 2000). Among the many subtypes of PKC, protein kinase C-β (PKCβ) is stimulated by HG (Hayashi et al., 2017). Furthermore, PKCβ is involved in many heart diseases (Mochly-Rosen et al., 2012; Newton et al., 2016). Here, we measured the levels of phosphorylated and total PKC and ERK in HG (33 mM)-induced myocytes treated with or without A438079. As shown in Figures 6A,B, the levels of phosphorylated PKCβ and ERK were increased upon HG stimulation and were remarkably reduced after the addition of A438079, indicating that the P2X7R, an upstream regulator, may adjust PKCβ and ERK activation in HG-induced myocytes.

We pretreated cells with inhibitors of these kinases (LY317615, an inhibitor of PKCβ, or PD98059, an inhibitor of ERK) for 1 h and then incubated them with HG to further elucidate the relationship between PKCβ and ERK. After the addition of PKCβ and ERK inhibitors, the expression of the P2X7R did not change upon HG stimulation (Figures 6C,D). In contrast, LY317615 not only inhibited PKCβ activity but also reduced the activity of ERK (Figures 6C,E). However, PD98059 did not affect P2X7R and phospho-PKCβ activation (Figures 6C,F). Additionally, the levels of the molecular markers of cardiac hypertrophy, fibrosis, and apoptosis (such as MyHC, Col-1, MMP-9, TGF-β, Bax, Bcl-2, and Caspase-3) were decreased by PD98059 or LY317615 (Figure 6G). Thus, the activation of PKCβ and ERK was involved in HG-induced myocardial injury.

## Discussion

Cardiovascular disease remains one of the main causes of death worldwide. In the present study, we systematically revealed the novel role of the P2X7R in DCM. The P2X7R was upregulated in the diabetic hearts and in HG-treated cardiomyocytes. The inhibition or knockout of the P2X7R significantly mitigated cardiac damage, including cardiomyocyte fibrosis, apoptosis, and hypertrophy, *in vivo* and *in vitro*. We confirmed that the suppression of P2X7R inhibited PKCβ and ERK activation and then decreased the hallmarks of HG-induced myocardial remodeling. In addition, PKCβ was identified as an upstream molecule that regulates the activation of ERK in this process. Figure 6H summarizes the graphical abstract.

Diabetic cardiomyopathy is caused by prolonged diabetes (Aneja et al., 2008). Diabetes mainly changes the structure and function of the myocardium. Hyperglycemia not only causes morphological changes in the myocardium, such as cardiomyocyte hypertrophy, but also affects the myocardial structure and induces interstitial and perivascular fibrosis, eventually leading to heart failure (Kannel and McGee, 1979; Mizushige et al., 2000; Singh et al., 2008; Bugger and Abel, 2009, 2014). Reversing these detrimental processes will improve the cardiac function of patients with diabetes mellitus.

The P2X7R was first cloned from a rat brain (Surprenant et al., 1996). With advances in research, the P2X7R was subsequently detected in all types of cells *in vivo* (Collo et al., 1997; Rassendren et al., 1997; Fountain and Burnstock, 2009). In addition, P2X7R expression was not obvious in the kidney under physiological conditions. However, in abnormal cases, such as diabetes and hypertension, P2X7R expression was significantly increased in rats (Vonend et al., 2004). Moreover, increased P2X7R activity may contribute to the pathogenesis of diabetic nephropathy, and P2X7R inhibition reduces renal macrophage accumulation and contributes to reducing the high prevalence of kidney disease observed in patients with diabetes (Kreft et al., 2016; Menzies et al., 2017). Thus, the P2X7R exerts detrimental effects and is associated with organ damage caused by high sugar levels. Additionally, the P2X7R also exerts important physiological and pathological effects on the cardiovascular system (Burnstock, 2017). For example, stimulation of the P2X7R regulates pro-inflammatory responses in endothelial cells (Sathanoori et al., 2015) and induces autoimmunity in individuals with dilated cardiomyopathy (Martinez et al., 2015). In the present study, the cardiac function of the STZ-induced groups was significantly worse, based on the echocardiography results (Tables 2, 3). Cardiac function and corresponding parameters were significantly improved in P2X7R knockout mice or in mice administered the P2X7R inhibitor. Meanwhile, P2X7R expression was substantially upregulated, and subsequently increased the expression of β-Myhc, Collagen I, and TGF-β in both animal models and cells exposed to HG, whereas P2X7R blockade significantly reversed myocardial fibrosis and reduced hypertrophy, indicating that high P2X7R expression is involved in cardiac remodeling by modulating fibrosis and hypertrophy in subjects with DCM. Notably, cell apoptosis contributes to the development of cardiac remodeling (Wang et al., 2014), and reducing the apoptosis of cardiac tissue may be an effective therapeutic strategy. Here, Caspase 3 and Bax levels increased, whereas the level of the antiapoptotic protein Bcl-2 was decreased, similar to the results from the TUNEL assay. However, the addition of the P2X7R inhibitor (A438079) or P2X7R**^–/–^** reversed these aberrant changes. Collectively, the P2X7R is involved in the process of fibrosis, hypertrophy, and apoptosis in heart tissues of diabetic mice, suggesting that the inhibition of P2X7R expression to prevent cardiac remodeling will be an effective cardioprotective strategy for abolishing DCM. Interestingly, the change in P2X7R expression only occurred in cardiomyocytes, but not in cultured rat primary fibroblasts (Supplementary Figure 1D), which is supported by the results showing that fibroblasts in the heart mostly mediate fibrosis through P2Y receptors (Certal et al., 2017). This phenomenon indicates that cardiomyocytes are the main sources of P2X7R expression leading to cardiac remodeling in DCM.

Based on the P2X7R structure, the N terminus contains a consensus PKC phosphorylation site (Boue-Grabot et al., 2000). Furthermore, P2X7R activation promotes calcium influx, which activates the PKC protein (Surprenant et al., 1996). Thus, various phenomena indicate a link between the P2X7R and PKC protein. Naturally, the PKC protein becomes the focus because of its important function in cells (Antal et al., 2015). Given its different structures, PKC is divided into three subfamilies. PKCβ is one of the conventional PKCs detected in heart tissue. Based on ample evidence, the PKCβ isoform is overactivated in the hearts of rats with diabetes mellitus (Disatnik et al., 1994; Connelly et al., 2009; Liu et al., 2012). Mice with specific overexpression of PKCβ in the myocardium exhibit left ventricular hypertrophy, cardiac myocyte necrosis, and multifocal fibrosis (Wakasaki et al., 1997). Therefore, we investigated whether the activation of PKCβ is involved in the mechanism by which the P2X7R in cardiomyocytes regulates hyperglycemia/STZ-induced cardiac remodeling. As expected, the phosphorylation of PKCβ was increased when H9c2 cells were stimulated with HG (Figure 6A). On the other hand, P2X7R inhibition decreased the phosphorylation of PKCβ (Figure 6A). We explored the possible downstream targets of PKCβ to further mechanistically investigate the effect of PKCβ on diabetes. Extracellular signal-regulated kinases (ERK) are an important subfamily of mitogen-activated protein kinases that control a broad range of cellular activities and physiological processes (Lu and Xu, 2006; Keshet and Seger, 2010; RoskoskiJr., 2012). P2X7R stimulation in HEK-293 cells leads to the activation of ERK1 and ERK2 independent of Ca^2+^ influx (Amstrup and Novak, 2003), and ERK1/2 are activated by similar PKC-dependent signaling pathways (Bradford and Soltoff, 2002). In the present study, the P2X7R inhibitor A438079 suppressed phospho-PKCβ activity and decreased phospho-ERK activity in HG-induced H9c2 cells (Figures 6A,B), indicating that P2X7R is a critical upstream molecule of PKCβ and ERK. However, when the inhibitor of PKCβ was added to the cells, P2X7R activity was not affected, but ERK phosphorylation was reduced (Figure 6C). Interestingly, neither PKCβ nor P2X7R activity was altered by the administration of the ERK inhibitor PD98059 (Figure 6C). Thus, PKCβ mediates ERK activation initiated by the P2X7R in HG-induced H9c2 cells. Similarly, the inhibition of PKC and ERK abolished HG-induced cell hypertrophy, fibrosis, and apoptosis (Figure 6G). Taken together, the P2X7R, which is upstream of PKC, subsequently regulates the ERK pathway to participate in the pathogenesis of DCM (Figure 6H).

In general, we described the role of the P2X7R in DCM using its specific inhibitors and P2X7R**^–/–^** mice. P2X7R inhibition or deficiency reduces myocardial hypertrophy, fibrosis, and apoptosis to subsequently improve cardiac function. Moreover, we further clarified that the P2X7R/PKCβ/ERK pathway is involved in the process and provided a new target for the treatment of DCM.

## Data Availability Statement

The original contributions presented in the study are included in the article/Supplementary Material, further inquiries can be directed to the corresponding author/s.

## Ethics Statement

The animal study was reviewed and approved by the Committee on Animal Care of Wenzhou Medical University.

## Author Contributions

SH wrote the manuscript. SH and WW conceived, designed, and analyzed the data. SH, WW, and LL researched the data and performed the animal experiments. TW, YZ, and YL performed the cell experiments. WH reviewed and edited the manuscript. YW and ZH designed and supervised the study. All authors read and approved the final manuscript.

## Conflict of Interest

The authors declare that the research was conducted in the absence of any commercial or financial relationships that could be construed as a potential conflict of interest.

## Publisher’s Note

All claims expressed in this article are solely those of the authors and do not necessarily represent those of their affiliated organizations, or those of the publisher, the editors and the reviewers. Any product that may be evaluated in this article, or claim that may be made by its manufacturer, is not guaranteed or endorsed by the publisher.

## References

[B1] Amstrup J. Novak I. (2003). P2X7 receptor activates extracellular signal-regulated kinases ERK1 and ERK2 independently of Ca2+ influx. *Biochem. J.* 374 (Pt 1) 51–61. 10.1042/BJ20030585 12747800 PMC1223572

[B2] Aneja A. Tang W. H. Bansilal S. Garcia M. J. Farkouh M. E. (2008). Diabetic cardiomyopathy: insights into pathogenesis, diagnostic challenges, and therapeutic options. *Am. J. Med.* 121 748–757. 10.1016/j.amjmed.2008.03.046 18724960

[B3] Antal C. E. Hudson A. M. Kang E. Zanca C. Wirth C. Stephenson N. L. (2015). Cancer-associated protein kinase C mutations reveal kinase’s role as tumor suppressor. *Cell* 160 489–502. 10.1016/j.cell.2015.01.001 25619690 PMC4313737

[B4] Boue-Grabot E. Archambault V. Seguela P. (2000). A protein kinase C site highly conserved in P2X subunits controls the desensitization kinetics of P2X(2) ATP-gated channels. *J. Biol. Chem.* 275 10190–10195. 10.1074/jbc.275.14.10190 10744703

[B5] Bradford M. D. Soltoff S. P. (2002). P2X7 receptors activate protein kinase D and p42/p44 mitogen-activated protein kinase (MAPK) downstream of protein kinase C. *Biochem. J.* 366 (Pt 3) 745–755. 10.1042/BJ20020358 12057008 PMC1222820

[B6] Bugger H. Abel E. D. (2009). Rodent models of diabetic cardiomyopathy. *Dis. Model. Mech.* 2 454–466. 10.1242/dmm.001941 19726805

[B7] Bugger H. Abel E. D. (2014). Molecular mechanisms of diabetic cardiomyopathy. *Diabetologia* 57 660–671. 10.1007/s00125-014-3171-6 24477973 PMC3969857

[B8] Burnstock G. (2017). Purinergic signaling in the cardiovascular system. *Circ. Res.* 120 207–228. 10.1161/CIRCRESAHA.116.309726 28057794

[B9] Burnstock G. Knight G. E. (2004). Cellular distribution and functions of P2 receptor subtypes in different systems. *Int. Rev. Cytol.* 240 31–304. 10.1016/S0074-7696(04)40002-3 15548415

[B10] Certal M. Vinhas A. Barros-Barbosa A. Ferreirinha F. Costa M. A. Correia-de-Sa P. (2017). ADP-induced Ca(2+) signaling and proliferation of rat ventricular myofibroblasts depend on phospholipase C-linked TRP channels activation within lipid rafts. *J. Cell. Physiol.* 232 1511–1526. 10.1002/jcp.25656 27755650

[B11] Collo G. Neidhart S. Kawashima E. Kosco-Vilbois M. North R. A. Buell G. (1997). Tissue distribution of the P2X7 receptor. *Neuropharmacology* 36 1277–1283. 10.1016/s0028-3908(97)00140-8 9364482

[B12] Connelly K. A. Kelly D. J. Zhang Y. Prior D. L. Advani A. Cox A. J. (2009). Inhibition of protein kinase C-beta by ruboxistaurin preserves cardiac function and reduces extracellular matrix production in diabetic cardiomyopathy. *Circ. Heart Fail.* 2 129–137. 10.1161/CIRCHEARTFAILURE.108.765750 19808328

[B13] Disatnik M. H. Buraggi G. Mochly-Rosen D. (1994). Localization of protein kinase C isozymes in cardiac myocytes. *Exp. Cell Res.* 210 287–297. 10.1006/excr.1994.1041 8299726

[B14] Forbes J. M. Cooper M. E. (2013). Mechanisms of diabetic complications. *Physiol. Rev.* 93 137–188. 10.1152/physrev.00045.2011 23303908

[B15] Fountain S. J. Burnstock G. (2009). An evolutionary history of P2X receptors. *Purinergic Signal.* 5 269–272. 10.1007/s11302-008-9127-x 19015952 PMC2717308

[B16] Hayashi T. Shibata H. Kurihara I. Yokota K. Mitsuishi Y. Ohashi K. (2017). High glucose stimulates mineralocorticoid receptor transcriptional activity through the protein kinase C beta signaling. *Int. Heart J.* 58 794–802. 10.1536/ihj.16-649 28966330

[B17] Huang C. Yu W. Cui H. Wang Y. Zhang L. Han F. (2014). P2X7 blockade attenuates mouse liver fibrosis. *Mol. Med. Rep.* 9 57–62. 10.3892/mmr.2013.1807 24247209

[B18] Huang Z. Wang C. Wei L. Wang J. Fan Y. Wang L. (2008). Resveratrol inhibits EMMPRIN expression via P38 and ERK1/2 pathways in PMA-induced THP-1 cells. *Biochem. Biophys. Res. Commun.* 374 517–521. 10.1016/j.bbrc.2008.07.058 18647594

[B19] Kannel W. B. McGee D. L. (1979). Diabetes and cardiovascular disease. The Framingham study. *JAMA* 241 2035–2038. 10.1001/jama.1979.03290450033020 430798

[B20] Keshet Y. Seger R. (2010). The MAP kinase signaling cascades: a system of hundreds of components regulates a diverse array of physiological functions. *Methods Mol. Biol.* 661 3–38. 10.1007/978-1-60761-795-2_1 20811974

[B21] Kreft E. Kowalski R. Jankowski M. Szczepanska-Konkel M. (2016). Renal vasculature reactivity to agonist of P2X7 receptor is increased in streptozotocin-induced diabetes. *Pharmacol. Rep.* 68 71–74. 10.1016/j.pharep.2015.06.140 26721355

[B22] Liu Y. Lei S. Gao X. Mao X. Wang T. Wong G. T. (2012). PKCbeta inhibition with ruboxistaurin reduces oxidative stress and attenuates left ventricular hypertrophy and dysfunction in rats with streptozotocin-induced diabetes. *Clin. Sci.* 122 161–173. 10.1042/CS20110176 21892921

[B23] Lu Z. Xu S. (2006). ERK1/2 MAP kinases in cell survival and apoptosis. *IUBMB Life* 58 621–631. 10.1080/15216540600957438 17085381

[B24] Martinez C. G. Zamith-Miranda D. da Silva M. G. Ribeiro K. C. Brandao I. T. Silva C. L. (2015). P2x7 purinergic signaling in dilated cardiomyopathy induced by auto-immunity against muscarinic M2 receptors: autoantibody levels, heart functionality and cytokine expression. *Sci. Rep.* 5:16940. 10.1038/srep16940 26592184 PMC4655336

[B25] Menzies R. I. Booth J. W. R. Mullins J. J. Bailey M. A. Tam F. W. K. Norman J. T. (2017). Hyperglycemia-induced renal P2X7 receptor activation enhances diabetes-related injury. *EBioMedicine* 19 73–83. 10.1016/j.ebiom.2017.04.011 28434946 PMC5440600

[B26] Mezzaroma E. Toldo S. Farkas D. Seropian I. M. Van Tassell B. W. Salloum F. N. (2011). The inflammasome promotes adverse cardiac remodeling following acute myocardial infarction in the mouse. *Proc. Natl. Acad. Sci. U.S.A.* 108 19725–19730. 10.1073/pnas.1108586108 22106299 PMC3241791

[B27] Mizushige K. Yao L. Noma T. Kiyomoto H. Yu Y. Hosomi N. (2000). Alteration in left ventricular diastolic filling and accumulation of myocardial collagen at insulin-resistant prediabetic stage of a type II diabetic rat model. *Circulation* 101 899–907. 10.1161/01.cir.101.8.899 10694530

[B28] Mochly-Rosen D. Das K. Grimes K. V. (2012). Protein kinase C, an elusive therapeutic target? *Nat. Rev. Drug Discov.* 11 937–957. 10.1038/nrd3871 23197040 PMC3760692

[B29] Newton A. C. Antal C. E. Steinberg S. F. (2016). Protein kinase C mechanisms that contribute to cardiac remodelling. *Clin. Sci.* 130 1499–1510. 10.1042/CS20160036 27433023 PMC5024564

[B30] Ofstad A. P. (2016). Myocardial dysfunction and cardiovascular disease in type 2 diabetes. *Scand. J. Clin. Lab. Invest.* 76 271–281. 10.3109/00365513.2016.1155230 27071642

[B31] Ogurtsova K. da Rocha Fernandes J. D. Huang Y. Linnenkamp U. Guariguata L. Cho N. H. (2017). IDF Diabetes Atlas: global estimates for the prevalence of diabetes for 2015 and 2040. *Diabetes Res. Clin. Pract.* 128 40–50. 10.1016/j.diabres.2017.03.024 28437734

[B32] Peng K. Liu L. Wei D. Lv Y. Wang G. Xiong W. (2015). P2X7R is involved in the progression of atherosclerosis by promoting NLRP3 inflammasome activation. *Int. J. Mol. Med.* 35 1179–1188. 10.3892/ijmm.2015.2129 25761252 PMC4380202

[B33] Rassendren F. Buell G. N. Virginio C. Collo G. North R. A. Surprenant A. (1997). The permeabilizing ATP receptor, P2X7. Cloning and expression of a human cDNA. *J. Biol. Chem.* 272 5482–5486. 10.1074/jbc.272.9.5482 9038151

[B34] Riteau N. Gasse P. Fauconnier L. Gombault A. Couegnat M. Fick L. (2010). Extracellular ATP is a danger signal activating P2X7 receptor in lung inflammation and fibrosis. *Am. J. Respir. Crit. Care Med.* 182 774–783. 10.1164/rccm.201003-0359OC 20522787

[B35] Roskoski R. Jr. (2012). ERK1/2 MAP kinases: structure, function, and regulation. *Pharmacol. Res.* 66 105–143. 10.1016/j.phrs.2012.04.005 22569528

[B36] Sathanoori R. Sward K. Olde B. Erlinge D. (2015). The ATP receptors P2X7 and P2X4 modulate high glucose and palmitate-induced inflammatory responses in endothelial cells. *PLoS One* 10:e0125111. 10.1371/journal.pone.0125111 25938443 PMC4418812

[B37] Singh V. P. Le B. Khode R. Baker K. M. Kumar R. (2008). Intracellular angiotensin II production in diabetic rats is correlated with cardiomyocyte apoptosis, oxidative stress, and cardiac fibrosis. *Diabetes* 57 3297–3306. 10.2337/db08-0805 18829990 PMC2584136

[B38] Soetikno V. Sari F. R. Sukumaran V. Lakshmanan A. P. Mito S. Harima M. (2012). Curcumin prevents diabetic cardiomyopathy in streptozotocin-induced diabetic rats: possible involvement of PKC-MAPK signaling pathway. *Eur. J. Pharm. Sci.* 47 604–614. 10.1016/j.ejps.2012.04.018 22564708

[B39] Stachon P. Heidenreich A. Merz J. Hilgendorf I. Wolf D. Willecke F. (2017). P2X7 deficiency blocks lesional inflammasome activity and ameliorates atherosclerosis in mice. *Circulation* 135 2524–2533. 10.1161/CIRCULATIONAHA.117.027400 28377486

[B40] Surprenant A. Rassendren F. Kawashima E. North R. A. Buell G. (1996). The cytolytic P2Z receptor for extracellular ATP identified as a P2X receptor (P2X7). *Science* 272 735–738. 10.1126/science.272.5262.735 8614837

[B41] Vonend O. Turner C. M. Chan C. M. Loesch A. Dell’Anna G. C. Srai K. S. (2004). Glomerular expression of the ATP-sensitive P2X receptor in diabetic and hypertensive rat models. *Kidney Int.* 66 157–166. 10.1111/j.1523-1755.2004.00717.x 15200422

[B42] Wakasaki H. Koya D. Schoen F. J. Jirousek M. R. Ways D. K. Hoit B. D. (1997). Targeted overexpression of protein kinase C beta2 isoform in myocardium causes cardiomyopathy. *Proc. Natl. Acad. Sci. U.S.A.* 94 9320–9325. 10.1073/pnas.94.17.9320 9256480 PMC23178

[B43] Wang H. J. Wang W. Cornish K. G. Rozanski G. J. Zucker I. H. (2014). Cardiac sympathetic afferent denervation attenuates cardiac remodeling and improves cardiovascular dysfunction in rats with heart failure. *Hypertension* 64 745–755. 10.1161/HYPERTENSIONAHA.114.03699 24980663 PMC4162756

[B44] Wang J. Song Y. Wang Q. Kralik P. M. Epstein P. N. (2006). Causes and characteristics of diabetic cardiomyopathy. *Rev. Diabet. Stud.* 3 108–117. 10.1900/RDS.2006.3.108 17487334 PMC1783586

[B45] Zhao X. Zhu X. Zhang H. Zhao W. Li J. Shu Y. (2012). Prevalence of diabetes and predictions of its risks using anthropometric measures in southwest rural areas of China. *BMC Public Health* 12:821. 10.1186/1471-2458-12-821 22998969 PMC3549931

