# Supplementary material for: P2X7 Receptor Deficiency Ameliorates STZ-induced Cardiac Damage and Remodeling Through PKCβ and ERK
Source: Front Cell Dev Biol. 2021 Jul 29;9:692028. doi: 10.3389/fcell.2021.692028 (PMC8358615; doi:10.3389/fcell.2021.692028)
Supplement: Supplementary file 1 [file Data_Sheet_1.docx]

Supplementary Material

**Supplemental Information titles and legends**

**Figure S1** western blot analysis of P2X7R expression in H9c2 cells treated with a high concentration of glucose (33 mM) for different periods (A-B). H9c2 cells and primary cardiomyocytes were pretreated with the vehicle (DMSO, 1 µl) or A438079 (10 μM) for 1 h and then incubated with HG (33 mM) for 24 h. P2X7R expression levels were measured using western blot and real-time PCR analyses (C, D). Primary cardiac fibroblasts were incubated with HG (33 mM) for different times. Western blot and real-time PCR analyses of P2X7R expression (E-F) in primary cardiac fibroblasts. Weekly weight and fasting blood glucose levels of each group of C57BL/6 mice (G-H). (Data from three independent experiments were analyzed: * p＜0.05, ** p＜0.01, and ***p＜0.001 compared with the Ctrl; # p<0.05, ##p< 0.01, and ###p<0.001 compared with HG alone.)

**Figure S2**

The respective statistical charts of the data presented in Fig. 4H (A and C) and Fig. 4I (B and D).
