# Supplementary figures and images for: P2X7 Receptor Deficiency Ameliorates STZ-induced Cardiac Damage and Remodeling Through PKCβ and ERK
Source: Front Cell Dev Biol. 2021 Jul 29;9:692028. doi: 10.3389/fcell.2021.692028 (PMC8358615; doi:10.3389/fcell.2021.692028)

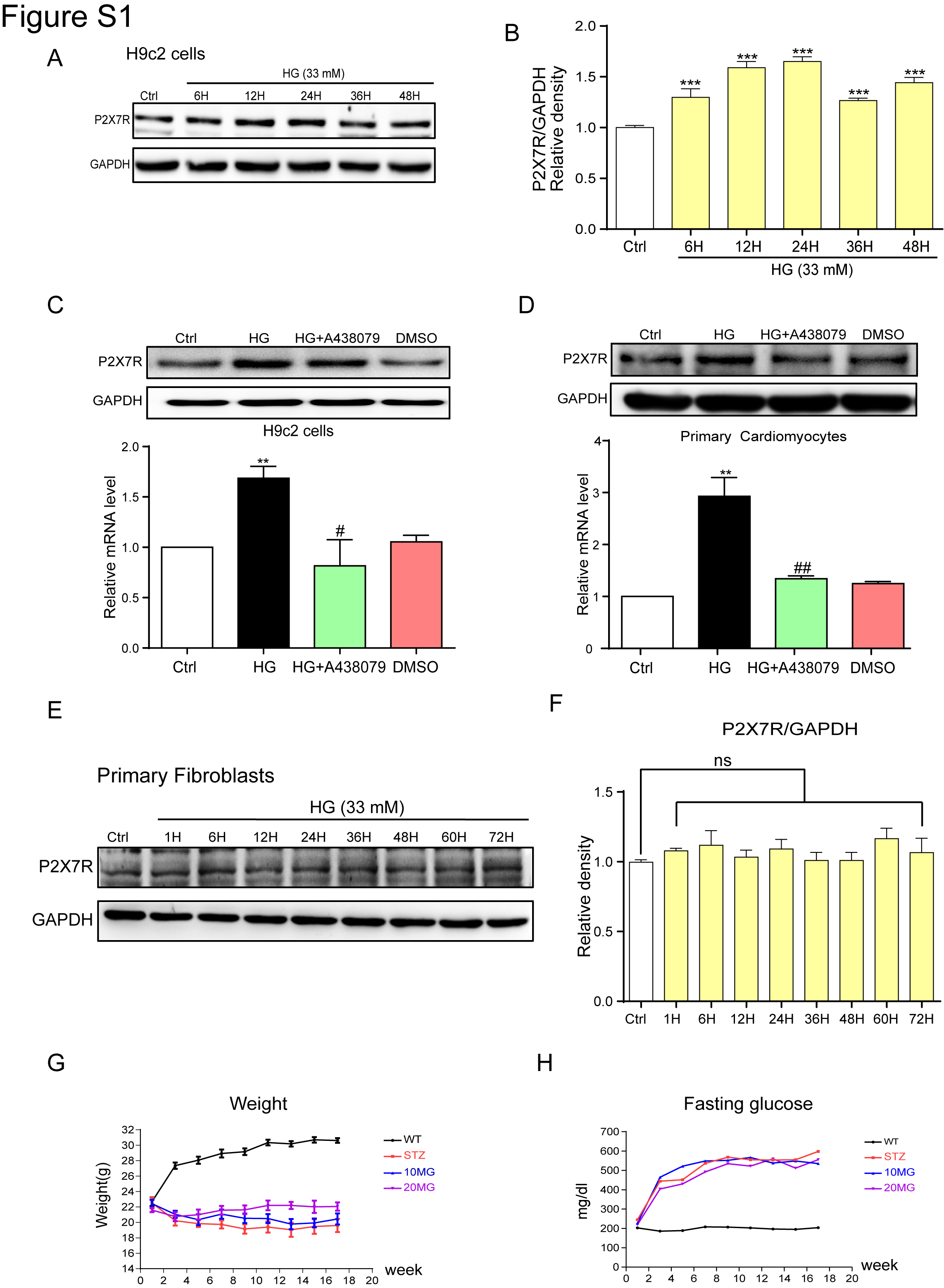

Supplement: Supplementary file 2 [file Image_1.TIF]

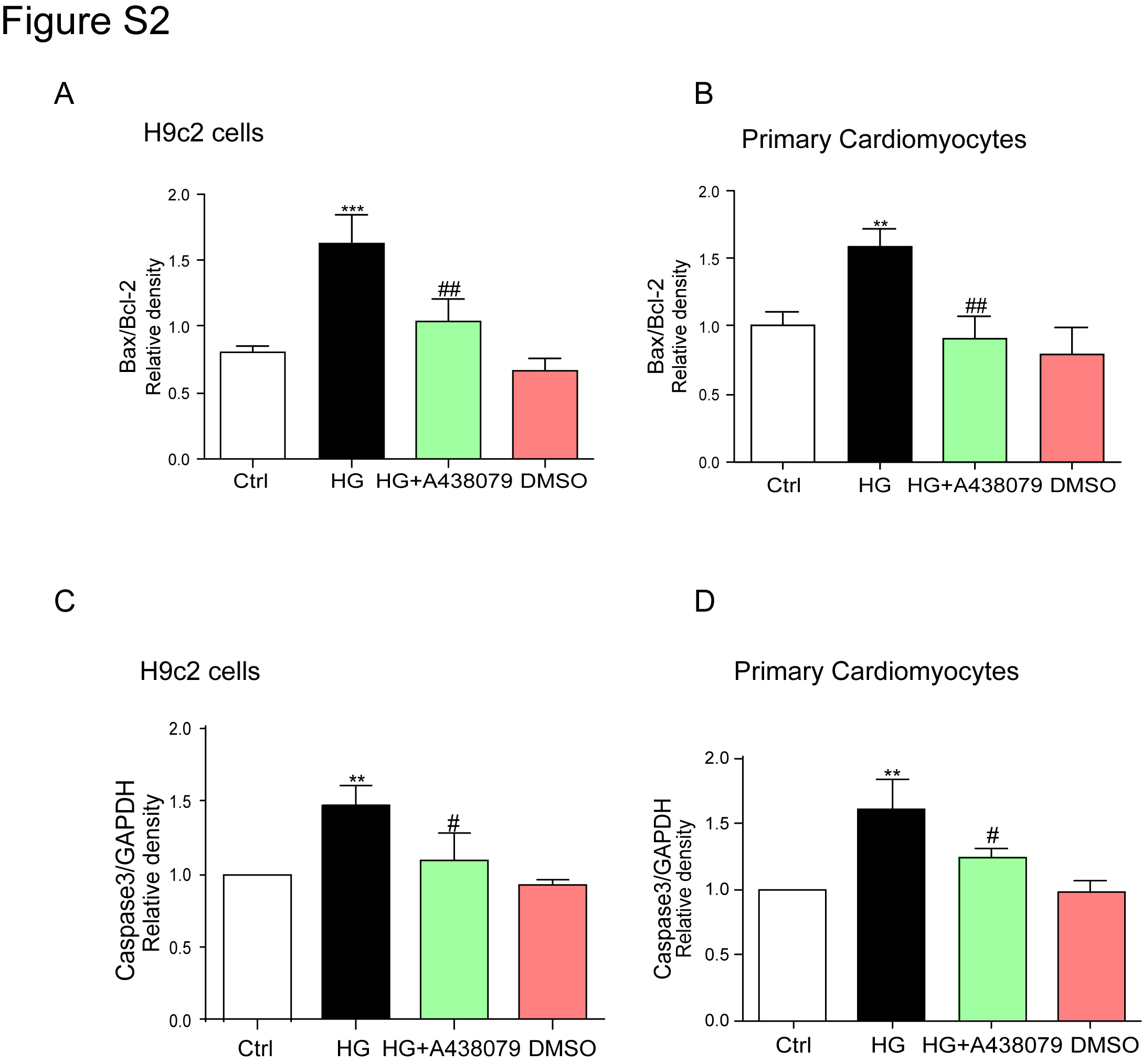

Supplement: Supplementary file 3 [file Image_2.TIF]
